# Farm management and breeding-related animal movement associated with African swine fever risk in smallholder pig farms in West Timor, Indonesia

**DOI:** 10.3389/fvets.2026.1907997

**Published:** 2026-09-09

**Authors:** Petrus Malo Bulu, Ewaldus Wera, Agustinus Paga

**Affiliations:** 1 Animal Health Study Program, Kupang State Agricultural Polytechnic (Politeknik Pertanian Negeri Kupang), Kupang, Indonesia; 2 Animal Feed Technology Study Program, Kupang State Agricultural Polytechnic (Politeknik Pertanian Negeri Kupang), Kupang, Indonesia

**Keywords:** African swine fever, animal movement, biosecurity, farm managements, risk factors, smallholder pig farms, West Timor

## Abstract

**Background:**

*African Swine Fever* (ASF) is a highly contagious transboundary animal disease that has led to substantial economic losses in smallholder pig production systems around the world. Some of the possible explanations for why ASF transmission still occurs in Indonesia, particularly West Timor, are traditional husbandry practices and poor biosecurity implementations. This study aimed to identify potential pig farm-level risk factors for ASF in smallholder farming systems of West Timor.

**Materials and methods:**

A cross-sectional study was carried out on 300 smallholder pig farms. Structured questionnaires and field observations on farm management, animal movement patterns, breeding, feeding management, and biosecurity implementation were employed for data collection. Associations with the occurrence of ASF were characterized using crude odds ratios (OR) by performing univariable analysis and were further evaluated using multivariable logistic regression analysis.

**Results:**

Multiple variables were significantly linked with the occurrence of ASF. In the multivariable model, farms that used breeding boars from outside the village or district were significantly more likely to be infected with ASF (OR = 3.94; 95% CI: 2.01–7.71; *p* < 0.001). High risk factors: introduction of pigs in the last 12 months (OR = 2.86; 95% CI:1.47–5.55; *p* = 0·002), contact with other pigs (OR = 3.12; 95% CI:1·61–6·03; *p* < 0·001) and swill feeding (OR = 2·41; 95% CI:1·27–4 ·56: *p* = 0 according to Fisher’s extract) were also independently associated with high risks for ASF infection. The final model performed well, with a Nagelkerke R^2^ of 0.41 and an accuracy of classification of 78.6%.

**Conclusion:**

The study highlights the significant role that informal animal movement networks, inter-farm pig contact, and poor feeding and biosecurity practices may play in the transmission of *ASF* across West Timor. The spread of ASF in smallholder pig production systems can be minimized through the strengthening of movement control and improving farm-level biosecurity and feeding management practices.

## 1 Introduction

African swine fever (ASF) continues to threaten pig production worldwide, including Asia (1, 2). Limited biosecurity and frequent informal pig movements may facilitate virus transmission, yet evidence on farm-level risk factors in eastern Indonesia remains limited. The disease is characterized by high morbidity and mortality rates, severe economic losses, disruption of pig production systems, and significant impacts on food security and livelihoods, particularly in low- and middle-income countries dependent on smallholder pig farming systems (3, 4). Since an ideal ASF vaccine has not yet been achieved (5) and is not widely available, prevention and control of ASF rely heavily on strict biosecurity, early detection, movement control, and rapid outbreak response measures.

Over the last decade, ASF has spread rapidly across Africa, Europe, and Asia, becoming a major global veterinary concern. Following its introduction into Asia in 2018, the disease caused extensive outbreaks in several countries, including China, Vietnam, the Philippines, and Indonesia (6, 7). Indonesia officially reported ASF outbreaks in 2019, and since then the disease has continued to affect multiple provinces, including East Nusa Tenggara Province. The widespread distribution of ASF in Indonesia has caused substantial reductions in pig populations and severe socio-economic consequences for communities relying on pig farming as a source of income, savings, and cultural value.

In East Nusa Tenggara, particularly in West Timor, pig farming is predominantly practiced under traditional smallholder systems characterized by backyard production, simple housing structures, low biosecurity implementation, and frequent informal animal movement (8). Pigs play an important economic, cultural, and social role in local communities, where they are commonly used for traditional ceremonies, religious events, and household income generation (9). However, traditional smallholder pig production systems often create favorable conditions for African swine fever (ASF) transmission because pigs are typically raised under low-input management with limited biosecurity. Consequently, frequent pig-to-pig contact, informal movement of live pigs for trade and natural breeding, and limited quarantine practices create favorable conditions for the introduction and dissemination of ASFV between farms (10–12). Moreover, swill feeding remains common in many smallholder systems and has been identified as an important pathway for ASFV introduction when contaminated pork products are used as feed (3, 13). Furthermore, limited farmer awareness, inadequate hygiene practices, and restricted access to veterinary services may reduce the effectiveness of on-farm disease prevention, thereby increasing the susceptibility of smallholder pig farms to ASF occurrence (14).

Previous studies have identified several factors associated with ASF transmission, including movement of infected pigs (15), contaminated pork products (16), swill feeding (17), poor sanitation (18), and inadequate farm biosecurity (19, 20). Animal movement networks, particularly informal trade and exchange systems, are considered major factors associated with ASF occurrence in endemic areas. Similarly, swill feeding has repeatedly been linked to ASF outbreaks because contaminated pork products may contain infectious virus capable of surviving for prolonged periods in meat and food waste.

Although several studies have investigated ASF epidemiology globally, limited information is available regarding farm-level risk factors associated with ASF occurrence in traditional pig farming systems in eastern Indonesia. Understanding local transmission dynamics and management-related risk factors is essential for designing effective and context-specific disease control strategies. In smallholder settings such as West Timor, routine husbandry practices including breeding boar exchange, pig introduction, and inter-farm contact may function as important but often overlooked epidemiological pathways facilitating virus dissemination.

Therefore, this study aimed to identify risk factors associated with ASF occurrence in smallholder pig farms in West Timor, Indonesia. Specifically, the study evaluated the influence of animal movement, breeding management, feeding practices, and farm biosecurity implementation on ASF transmission. The findings are expected to provide epidemiological evidence supporting the development of risk-based ASF prevention and control strategies adapted to smallholder pig production systems in the region.

## 2 Materials and methods

### 2.1 Study area

**Figure 1:**
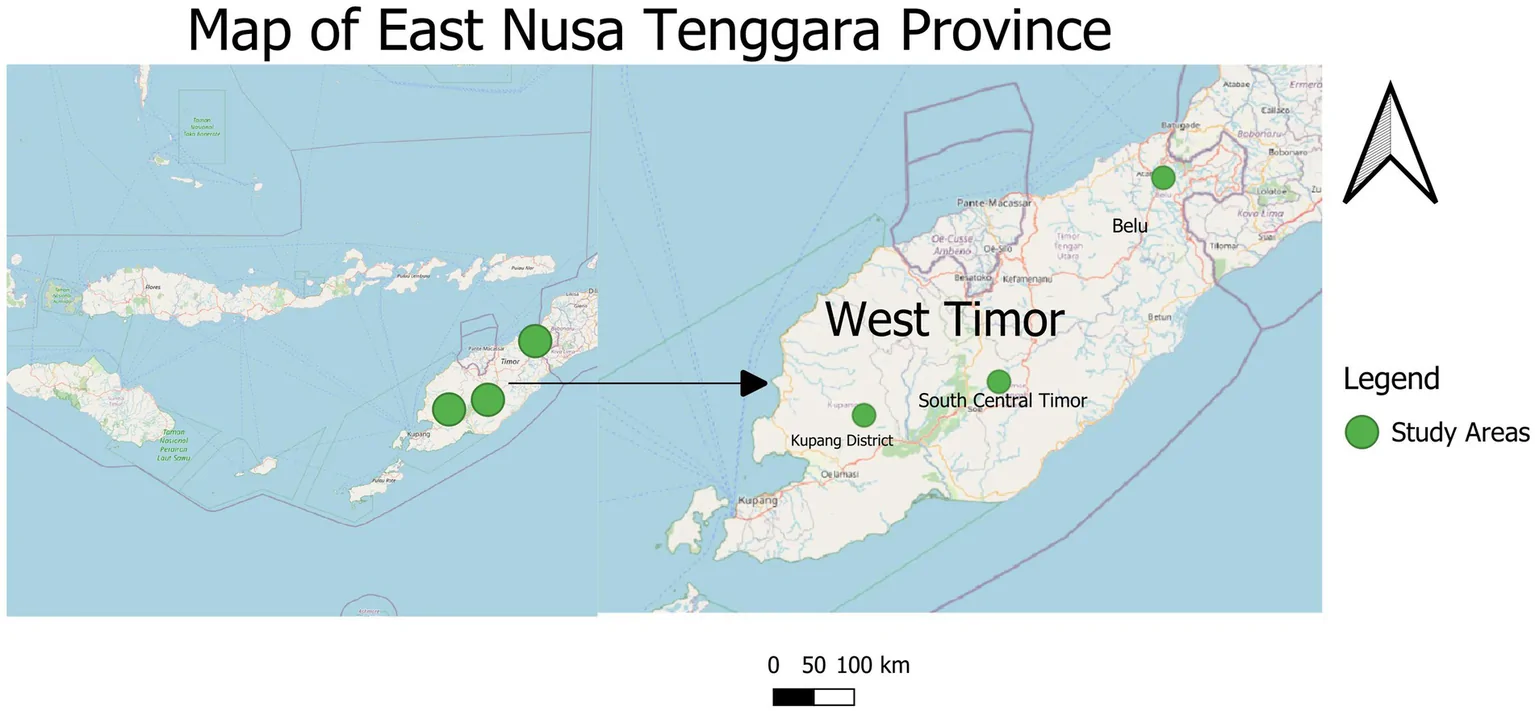
Map of East Nusa Tenggara Province, Indonesia, showing the study area in the West Timor region. Study locations are highlighted in green circles, including Kupang District, South Central Timor (Timor Tengah Selatan), and Belu. The map provides geographic context for the study area and includes labeled administrative boundaries, a legend, and a scale bar. Created by the authors using QGIS based on the study’s original geographic and study-site data.

The study was conducted in smallholder pig farming areas involving 3 districts (Kupang, South Central Timor and Belu) in West Timor- East Nusa Tenggara Province, Indonesia, a region characterized by traditional pig production systems and high dependence on pigs for socio-economic and cultural activities (Figure 1). Pig farming in this region is predominantly raised under backyard or semi-intensive systems with varying levels of biosecurity implementation. The study area has experienced recurrent ASF outbreaks since 2019, making it an appropriate setting for investigating farm-level risk factors associated with disease occurrence.

### 2.2 Study design

A cross-sectional observational study was carried out to investigate the association between on-farm management, animal movement patterns, breeding, feeding management, biosecurity implementation, and ASF occurrence. Farms were classified as ASF-positive if pigs had laboratory-confirmed African swine fever virus (ASFV) infection by real-time PCR or if ASF cases were officially confirmed by the District Veterinary Services during the 2023 outbreaks. Farms without laboratory-confirmed or officially reported ASF cases during the 2023 outbreak period were classified as ASF-negative.

### 2.3 Study population and sampling

The study population consisted of farmers who were either owners or managers of pigs in the selected districts of West Timor. A total of 300 pig farmers were recruited using purposive sampling and interviewed face-to-face. Each participant represented a single smallholder pig farm, resulting in a total of 300 farms included in the analysis.

Farmers were eligible if they were actively involved in pig farming during the study period, owned or managed at least one herd, resided in the selected study villages, provided informed consent, and were available at the time of the survey. Purposive sampling was adopted because the study aimed to identify potential risk factors for the introduction and spread of ASF in an ASF-free area rather than estimating disease prevalence. Accordingly, farms were deliberately selected to represent diverse pig management practices and pig movement patterns considered relevant to ASF risk assessment. The selection was independent of herd size, proximity to major roads, or previous access to livestock services.

### 2.4 Data Collection (Questionnaire development and interview procedures)

Data were collected from July to September 2023 in three selected districts in West Timor. The data were collected through face-to-face interviews with a structured questionnaire. The questionnaire included information regarding housing management systems, breeding practices, the origin of breeding boars, animal movement practices, farm biosecurity measures, and ASF occurrence status. The questionnaire was developed using the published literature and expert input. Subsequently, it was pilot-tested with 10 pig farmers (5 men and 5 women) in Kupang to assess its clarity, relevance, and administration time and to identify and resolve any ambiguities before the main survey. The questionnaire collected information on housing management, breeding practices, breeding boar origin (own farm, same village, or outside the village/district), pig introduction within the previous 12 months, contact with pigs from other farms, swill feeding, farm biosecurity, and ASF occurrence status. The dependent variable was ASF occurrence, classified as ASF-positive or ASF-negative. The independent variables included housing management systems, breeding systems involving natural mating practices, and the origin of breeding boars, categorized as own farm, same village, other villages, and other districts.

### 2.5 Data analysis

The collected data were entered into Microsoft Excel 2021 (Microsoft Inc., Redmond, WA) before being imported into IBM SPSS Statistics version 25.0 (IBM Corp., Armonk, NY, USA) for subsequent statistical analysis. Univariable analysis was performed using Pearson’s chi-square or Fisher’s exact test when expected cell counts were <5. Odds ratios (OR) and 95% confidence intervals (CI) were calculated to assess associations between potential risk factors and ASF occurrence (21). Candidate variables showing associations at *p* < 0.25 in the univariable analysis were considered for inclusion in the multivariable model. Variables with high collinearity (*r* > 0.8) were excluded. Village was included as a random effect to account for clustering. Two-way interactions between explanatory variables were evaluated. Model fit was assessed using the Hosmer-Lemeshow test and Nagelkerke *R*^2^ values (22).

Educational attainment was initially recorded in five categories. For regression analyses, education was recategorized into two groups: low education (no formal education, elementary school and junior high school) and higher education (senior high school and university) to increase statistical power and ensure adequate cell counts.

### 2.6 Ethical considerations

The study received ethical clearance from the Livestock, Marine and Fishery Research Ethics Committee (KEPPKP), Faculty of Animal Husbandry, Marine and Fishery Sciences, Nusa Cendana University (Ref. No. 0165/1.KT/KEPPKP/VIII/2026). Participation was voluntary, and written informed consent was obtained from all respondents before data collection. Confidentiality of farm-level information was maintained throughout the study.

## 3 Results

### 3.1 Farm characteristics

The study was performed on 300 smallholder pig farms. The majority of the farmers held junior high school (30.3%) or elementary school education (28.3%), and only 10.0% had university-level education. Natural mating was the leading breeding system (86.0%). As for breeding boars, Most farms used breeding boars from their own farms (42.7%), and 26.6% of the farms used breeding boars from outside the village or district (Table 1).

**Table 1:** Characteristics of smallholder pig farms in West Timor.

Variable	Category	Number of farms (*n*)	Percentage (%)
Educational level	No formal education	13	4.3
	Elementary school	85	28.3
	Junior high school	91	30.3
	Senior high school	81	27.0
	University	30	10.0
Breeding system	Natural mating	258	86.0
	Other breeding systems	42	14.0
Breeding boar origin	Own farm (internal)	128	42.7
	Same village	92	30.7
	Outside village/district	80	26.6
Pig introduction (last 12 months)	Yes	130	43.3
	No	170	56.7
Contact with pigs from other farms	Yes	125	41.7
	No	175	58.3
Swill feeding	Yes	131	43.7
	No	169	56.3
Routine sanitation/disinfection	Yes	159	53.0
	No	141	47.0
Visitor restriction	Yes	159	53.0
	No	141	47.0
Isolation pen available	Yes	146	48.7
	No	154	51.3

Overall, 43% of farms reported introducing pigs during the previous 12 months, while 41.7% had contact with pigs from other farms concerning animal movement.

Swill feeding was reported by 43.7% of pig farmers. About 53.0% of the farms had regular sanitation and disinfection, while about half (53.0%) had visitor restriction, and only 48.7% had an isolation pen for newly introduced or sick animals (Table 1).

### 3.2 Univariable risk factor analysis associated with ASF occurrence in West Timor

Univariable analysis identified several management, biosecurity, and animal movement variables significantly associated with ASF occurrence in smallholder pig farms in West Timor (Table 2). Variables associated with education level, breeding management, animal introduction, inter-farm contact, and biosecurity implementation demonstrated significant relationships with ASF positivity.

**Table 2:** Univariable risk analysis of factors associated with ASF occurrence in West Timor.

Variable	Category	ASF positive *n* (%)	OR	95% CI	*p* value
Breeding boar origin	Outside village/district	86 (67.19)	4.78	2.61–8.74	<0.001
	Own farm/internal (Reference)	24 (30.00)	1.00	Reference	–
Pig introduction within 12 months	Yes	112 (63.5)	3.78	2.33–6.14	<0.001
	No (Reference)	37 (31.5)	1.00	Reference	–
Contact with other pigs	Yes	112 (64.0)	4.23	2.58–6.92	<0.001
	No (Reference)	37 (29.6)	1.00	Reference	–
Swill feeding	Yes	107 (58.9)	3.66	2.26–5.92	<0.001
	No (Reference)	42 (41.1)	1.00	Reference	–
Routine sanitation/disinfection	No	46 (32.6)	0.26	0.16–0.43	–
	Yes (Reference)	103 (64.78)	1.00	Reference	–
Visitor restriction	No	48 (45.28)	0.48	0.29–0.78	–
	Yes (Reference)	101 (63.52)	1.00	Reference	–
Isolation pen availability	No	57 (37.01)	0.34	0.22–0.55	–
	Yes (Reference)	92 (63.01)	1.00	Reference	–
Educational level	Low education	91 (64.54)	3.17	1.98–5.08	<0.001
	Higher education (Reference)	58 (36.48)	1.00	Reference	–

Farmers with a lower educational background showed significantly higher ASF occurrence compared with farmers with university education (OR = 1.72, 95%CI = 0.93–3.18, *p* = 0,083). Similarly, farms implementing natural mating systems and using breeding boars originating from outside the farm demonstrated increased ASF risk (OR = 4.78, 95%CI = 2.61–8.74, *p* = <0.001. Animal movement variables, including pig introduction within the previous 12 months and contact with other pigs, were also significantly associated with ASF occurrence.

Several biosecurity-related practices showed protective effects against ASF transmission, including availability of perimeter fencing, adequate spacing between barns, routine sanitation and disinfection, separation of farrowing sows, and restriction of visitors and vehicles entering the farm area (Table 2).

### 3.3 Multivariable logistic regression analysis

The multivariable logistic regression analysis (Table 3) showed that several management and animal movement variables remained independently associated with ASF occurrence in smallholder pig farms in West Timor. Additionally, the use of breeding boars originating from outside the village or district showed the strongest association with ASF occurrence (OR = 3.94; 95% CI: 2.01–7.71; *p* < 0.001). This finding indicates that farms practicing external breeding boar exchange had nearly four times higher odds of ASF infection compared with farms relying on internal breeding boars. Besides, pig introduction within the previous 12 months was also significantly associated with ASF occurrence (OR = 2.86; 95% CI: 1.47–5.55; *p* = 0.002). Farms introducing new pigs were almost three times more likely to experience ASF infection, suggesting that introduction of animals without proper quarantine or health screening may contribute substantially to disease transmission. Similarly, farms reporting contact with other pigs demonstrated significantly increased ASF risk (OR = 3.12; 95% CI: 1.61–6.03; *p* < 0.001). This finding supports the hypothesis that direct or indirect contact between pigs from different farms enhances opportunities for virus dissemination within traditional pig production systems.

**Table 3:** Multivariable logistic regression analysis of risk factors associated with ASF occurrence in West Timor.

Variable	Category	OR	95% CI	*p* value
Breeding boar origin	Outside village/district vs. own farm/internal	3.94	2.01–7.71	<0.001
Pig introduction within 12 months	Yes vs. No	2.86	1.47–5.55	0.002
Contact with other pigs	Yes vs. No	3.12	1.61–6.03	<0.001
Swill feeding	Yes vs. No	2.41	1.27–4.56	0.007
Educational level	Low vs. higher education	1.72	0.93–3.18	0.083

Swill feeding practice also remained significantly associated with ASF occurrence in the final model (OR = 2.41; 95% CI: 1.27–4.56; *p* = 0.007). Farms utilizing swill feed had more than twice the odds of ASF infection compared with farms not practicing swill feeding. This finding is epidemiologically important because improperly treated swill may contain contaminated pork products capable of transmitting ASF virus.

Although a lower educational level showed increased odds of ASF occurrence in the crude analysis, the association was no longer statistically significant after adjustment in the multivariable model (OR = 1.72; 95% CI: 0.93–3.18; *p* = 0.083). This suggests that the effect of education may be mediated through farm management practices and biosecurity implementation rather than acting as an independent risk factor.

Overall, the multivariable model indicates that ASF transmission in West Timor is strongly influenced by informal pig movement, inter-farm animal contact, and inadequate feeding and biosecurity practices. These findings emphasize the need for risk-based control strategies focusing on movement management, quarantine implementation, and strengthening practical biosecurity measures adapted to smallholder farming systems.

## 4 Discussion

### 4.1 Farm characteristics

The characteristics of the farms included in this study reflect the typical smallholder pig production systems commonly found in West Timor. Most farmers had only primary or secondary education, while relatively few had attained university-level education. Similar educational profiles have been reported in smallholder pig production systems across Southeast Asia, where pig farming is predominantly managed as a traditional household livelihood rather than a specialized commercial enterprise (34, 35, 8). The predominance of natural mating and the frequent use of breeding boars sourced from outside the farm indicate the existence of informal animal contact networks that may facilitate disease transmission. Such management practices have been recognized as important contributors to the spread of infectious diseases in smallholder pig populations because they increase direct and indirect contact between animals from different farms (13, 23).

The study also revealed that animal movement and biosecurity practices remain major concerns in West Timor. Nearly half of the farms reported introducing pigs within the previous year, maintaining contact with pigs from other farms, or practicing swill feeding. Furthermore, only about half of the farms implemented routine sanitation and disinfection, visitor restrictions, or maintained isolation facilities. These findings suggest that biosecurity implementation remains suboptimal in many backyard production systems. Previous studies have consistently shown that low biosecurity standards, uncontrolled animal movement, and feeding of untreated food waste increase the risk of ASF introduction and spread within smallholder pig populations (14, 24). Therefore, improving farmer awareness and promoting practical, low-cost biosecurity measures adapted to local farming conditions are essential components of ASF prevention and control programs in West Timor.

### 4.2 Breeding boar movement and ASF transmission dynamics

This study identified external breeding boar utilization as the strongest factor associated with ASF occurrence in smallholder pig farms in West Timor. Farms using breeding boars from outside the village or district had nearly four times higher odds of ASF occurrence than farms relying on their own breeding boars. This finding is consistent with previous studies identifying the movement of live pigs, including breeding animals, as one of the most important pathways for ASF virus (ASFV) introduction and dissemination among farms (10, 12, 25–27).

Recent experimental studies provide biological support for this association. Friedrichs et al. (36) detected ASFV in boar semen and reproductive tissues following experimental infection, while Friedrichs et al. (36) and Maes et al. (37) reported that ASFV may persist in boar semen for several weeks after infection. These findings suggest that infected breeding boars could potentially disseminate the virus during natural mating.

In West Timor, natural mating is widely practiced and frequently involves moving breeding boars between farms without quarantine or veterinary supervision. Such movements create epidemiological links between farms and may facilitate both direct and indirect virus transmission. Similar observations have been reported in smallholder pig production systems in Africa and Asia (28). Given the high environmental stability of ASFV and its ability to spread through contaminated fomites and human activities (3, 11, 29), strengthening biosecurity and regulating breeding boar movement should be prioritized to reduce ASF risk in traditional pig production systems (10, 25, 26).

### 4.3 Pig introduction

Pig introduction within the previous 12 months was also significantly associated with ASF occurrence. Farms introducing pigs had almost three times higher odds of ASF infection compared with farms without recent pig introduction.

This finding is biologically plausible because asymptomatic or incubating pigs may introduce ASF virus into previously unaffected farms. In many smallholder systems, newly purchased pigs are frequently introduced directly into existing herds without quarantine or health assessment. Such conditions increase the likelihood of silent virus transmission before clinical signs become apparent.

Previous studies and systematic reviews have identified pig trading, introduction of purchased pigs, and uncontrolled movement of animals as major ASF risk factors worldwide (10, 30).

Contact with other pigs was another important factor associated with ASF positivity. Farms reporting inter-farm pig contact had more than three times higher odds of infection. This result indicates that direct and indirect pig-to-pig interactions facilitate ASF transmission within backyard farming systems. Informal pig contact networks are particularly difficult to monitor in smallholder settings where farms are geographically close, and biosecurity implementation is limited. Similar findings have been described in epidemiological studies emphasizing the role of contact networks and proximity between farms in ASF spread (10).

### 4.4 Swill feeding

Swill feeding practice also remained significantly associated with ASF occurrence in the multivariable model. Farms practicing swill feeding showed more than twice the odds of ASF infection compared with farms not using swill feed. Feeding pigs with untreated food waste remains a recognized mechanism through which ASFV may be introduced into susceptible herds. Numerous ASF outbreaks globally have been linked to feeding untreated food waste containing infected pork products (30).

### 4.5 Biosecurity implications

Although several biosecurity variables showed protective effects in the univariable analysis, including sanitation, visitor restriction, and isolation pen availability, these variables did not remain independently significant in the final multivariable model. Nevertheless, their epidemiological importance should not be underestimated.

Previous studies have consistently demonstrated that effective farm biosecurity reduces ASF introduction and spread (28, 31, 32). Poor sanitation, unrestricted visitor access, and inadequate disinfection practices have repeatedly been identified as major contributors to ASF outbreaks in domestic pig farms (10, 30, 33).

### 4.6 Study limitations

This study has some limitations in interpretation of the findings. Three limitations exist with this study; the first is that cross-sectional study designs provide no evidence of cause and effect between exposure and outcome variables. Second, some management information was based on farmer memory and subject to recall bias. Third, relatively little of the study was conducted on intensive commercial pig production systems; most of the study was focused on smallholder systems in West Timor.

It is important to note the limitations that were associated with this study, but nevertheless it provided valuable epidemiological evidence of the importance of animal movement and farm management practices in ASF transmission in traditional pig farming systems.

### 4.7 Future research directions

Research looking at ASF transmission should be integrated and include methodologies such as social network analysis of pig movement, community-level biosecurity interventions, longitudinal surveillance studies, and quantitative risk modeling.

These approaches will enhance our understanding of potential transmission pathways and therefore facilitate the process of elaborating sustainable mitigation interventions in smallholder production systems for ASF.

## 5 Conclusion

The findings indicate that animal movement and farm management practices were independently associated with ASF occurrence among smallholder pig farms in West Timor. External breeding boar use, pig introduction, inter-farm pig contact, and swill feeding were significant risk factors identified in the multivariable model. These findings support strengthening movement control, quarantine of newly introduced pigs, farm biosecurity, and safer feeding practices to reduce ASF risk in traditional smallholder production systems.

## Data Availability

The raw data supporting the conclusions of this article will be made available by the authors, without undue reservation.
